# The diverse genetic genotypes of *Bartonella* species circulating in rodents from Inner Mongolia, Northern China

**DOI:** 10.1371/journal.pntd.0011462

**Published:** 2023-06-29

**Authors:** Jianyun Li, Chenxi Zhang, Miao Lu, Yu Wang, Wen Wang, Fang Liu, Shaoqing Wu, Yang Liu, Mengguang Fan, Kun Li

**Affiliations:** 1 General Center for Disease Control and Prevention of Inner Mongolia Autonomous Region, Huhehot City, Inner Mongolia Autonomous Region, China; 2 Inner Mongolia Medical University, Huhehot City, Inner Mongolia Autonomous Region, China; 3 State Key Laboratory of Infectious Disease Prevention and Control, National Institute for Communicable Disease Control and Prevention, Chinese Center for Disease Control and Prevention, Changping District, Beijing City, China; 4 Inner Mongolia Agricultural University, Huhehot City, Inner Mongolia Autonomous Region, China; 5 Ulanqab Center for Disease Control and Prevention, Ulanqab City, Inner Mongolia Autonomous Region, China; 6 Baotou Medical College, Baotou City, Inner Mongolia Autonomous Region, China; Johns Hopkins University Bloomberg School of Public Health, UNITED STATES

## Abstract

*Bartonella* are generally recognized as zoonotic pathogens of mammals, including many rodent species. However, data on the genetic diversity of *Bartonella* in some regions are still absent in China. In this study, we collected rodent samples (*Meriones unguiculatus*, *Spermophilus dauricus*, *Eolagurus luteus*, and *Cricetulus barabensis*) from Inner Mongolia located in Northern China. The *Bartonella* were detected and identified by sequencing the *gltA*, *ftsZ*, ITS, and *groEL* genes in them. An overall 47.27% (52/110) positive rate was observed. This may be the first report that *M*. *unguiculatus* and *E*. *luteus* harbor *Bartonella*. Phylogenetic and genetic analysis on *gltA*, *ftsZ*, ITS, and *groEL* genes indicated that the strains were divided into seven distinct clades, suggesting the diverse genetic genotypes of *Bartonella* species in this area. Of those, Clade 5 meets the criteria for identification as a novel species based on gene sequence dissimilarity to known *Bartonella* species and herein we name it “*Candidatus* Bartonella mongolica”.

## Author summary

*Bartonella* spp. are emerging zoonotic causative agents distributed worldwide and they pose a significant threat to human health worldwide. In this study, the *Bartonella* carried by rodents in Inner Mongolia were detected and studied. The high positive rate (47.27%) and the remarkable genetic diversity (seven phylogenetic clades) of *Bartonella* in rodents indicate that rodent-borne *Bartonella* may potentially be exposed to local peoples.

## Introduction

The genus *Bartonella* belonging to family Bartonellaceae, order Rhizobiales (Hyphomicrobiales) represents a group of Gram-negative, facultative intracellular bacteria with worldwide distribution. To date, this genus is composed of at least 53 species, 7 subspecies, and some unnamed *Bartonella* spp. according to the National Center for Biotechnology Information database [1]. With the expanded sampling and improved detecting methods, novel species from all over the world are still being discovered. Many *Bartonella* species have been recognized as etiologic agents of emerging infectious diseases for both humans and domestic animals. Until 2021, at least 20 *Bartonella* species have been reported to infect humans [1,2], with the syndromes varying from mild and unspecific clinical signs to life-threatening symptoms [3]. First identified in 1905 by Alberto Leonardo Barton Thompson, *B*. *bacilliformis* transmitted by sandflies is proved to be the agent of Carrion’s disease with high lethality in the absence of adequate treatment [4]. As the most common *Bartonella* species infecting humans, *B*. *quintana* is the agent of trench fever mainly representing endocarditis and chronic bacteremia, while *B*. *henselae* causes cat scratch disease representing endocarditis, bacillary angiomatosis, and peliosis hepatis [5,6]. In the past decades, more *Bartonella* species have been described to infect humans, including *B*. *clarridgeiae*, *B*. *elizabethae*, *B*. *grahamii*, *B*. *koehlerae*, *B*. *kosoyi*, *B*. *melophagi*, *B*. *rochalimae*, *B*. *tamiae*, and *B*. *washoensis*, mainly manifesting with symptoms of fever, fatigue, and endocarditis [7–15]. Of those, *B*. *elizabethae*, *B*. *grahamii*, *B*. *kosoyi*, and *B*. *washoensis* are all hosted by rodents.

Epidemiological studies have demonstrated that *Bartonella* are mainly transmitted by hematophagous arthropods such as fleas and lice by their bites [16]. *Bartonella* species have been detected in multiple mammalian hosts including carnivores, rodents, bats, deer, and even marine animals [17–19]. In the past decades, a remarkable diversity of *Bartonella* has been reported in rodents, which were considered one of the major reservoir hosts. However, increasing research efforts in bats and other taxa might reveal similarly high diversity [18–21]. *Bartonella* infects the erythrocytes and endothelial cells of these animals, and then establishes persistent infections producing bacteremia that may last for months [22]. In some rodents, vertical transmission of *Bartonella* was also observed [22]. These characteristics strongly suggest that rodents play an important role in preserving and transmitting *Bartonella* in nature.

In recent years, lots of studies have been performed on molecular investigations of *Bartonella* in rodents from China. Up to 2021, multiple *Bartonella* species have been detected in various provinces including Heilongjiang, Shaanxi, Fujian, Xinjiang, Henan, Zhejiang, Yunnan, Guangdong, Hainan, and Qinghai Provinces, China [23–28]. However, some of the studies are preliminary and only one single short gene fragment was analyzed. Furthermore, in the Inner Mongolia Autonomous Region located in Northern China, a region of plague epidemic with an area of 1183, 000 km^2^, almost no studies on *Bartonella* have been performed except for one study reporting *Meriones unguiculatus* rats harboring *Bartonella tribocorum* and *B*. *grahamii* in this area [29]. To improve our knowledge on the diversity and epidemiology of *Bartonella* in China, we collected rodent samples in this region and studied the *Bartonella* bacteria in them.

## Methods

### Ethics declaration

This study protocol was approved by the Ethics Committee of the National Institute for Communicable Disease Control and Prevention, the Chinese CDC (Approval No. 2021–011), and the General Center for Disease Control and Prevention of the Inner Mongolia Autonomous Region. All the rodents were treated in a humane manner in accordance with the “Rules for Implementation of Laboratory Animal Medicine” from the National Health Commission, China. The studies in this manuscript adhere to the ARRIVE (Animal Research: Reporting of In Vivo Experiments) guidelines for the reporting of animal experiments.

### Sample collection and DNA extraction

In an ongoing plague surveillance project in 2021, rodents were live-trapped using fried food as baits in the desert steppe in three locations (Hunger, Baiyin Chaoketu, and Naomugeng) of Siziwang Banner (111.71°E, 41.53°N, near the China-Mongolia border), Ulanqab City, Inner Mongolia Autonomous Region, Northern China. The steel traps (150 mm×80 mm) were produced by Li’s Mousetrap Equipment Manufacturer in Guixi City, Jiangxi Province, China. Traps were checked for rodents each day and cages containing rodents were transported to the lab. After morphological identification [30], the rodents were anesthetized using pentobarbital sodium (40 mg/kg) to minimize their suffering and then sacrificed by cervical dislocation. The leftover liver tissues were then collected and stored at -20°C until DNA extraction. After being washed three times with Phosphate Buffered Saline (PBS) and ground, liver samples (25 mg) were then subjected to DNA extraction. The DNeasy Blood &Tissue kit (QIAGEN, Germany) was used to extract the DNA according to the manufacturer´s instructions. The eluted DNA samples were stored in a -80°C refrigerator until PCR detection of *Bartonella*.

### Molecular detection of *Bartonella* bacteria by amplifying *gltA* gene

As previously indicated, the primers forward BhCS871.p and reverse BhCS1137.n [31] were used for the detection of *Bartonella* amplifying a conserved region of the citrate synthase gene (*gltA*). PCR amplification was performed using a conventional Sensoquest PCR System LabCycler (Germany). Positive and negative controls were set in each PCR run. The PCR parameters are as follows: denaturing at 95°C for three minutes, 40 cycles of 95°C for one minute, 56°C for one minute, and 72°C for one minute. PCR amplification was completed after a final incubation at 72°C for 10 minutes. The PCR products were analyzed by electrophoresis on agarose gel and then observed under UV light. All the PCR products of the expected size (approximately 380 bp) were sent for DNA sequencing. The obtained *gltA* sequences, which were commonly used for the taxonomic identification of *Bartonella* species [32], were then analyzed by BLASTn (https://blast.ncbi.nlm.nih.gov/Blast.cgi?PROGRAM = blastn&PAGE_TYPE = BlastSearch&LINK_LOC = blasthome) to preliminarily determine their genetic similarity to known species. The DNA of *Bartonella* sp. we previously detected in Guizhou Province was used as the positive control [33] and ddH_2_O was used as the negative control.

### PCR amplification and genetic analysis of *ftsZ*, *groEL*, and ITS genes

Based on genetic analysis of the obtained *gltA* sequences, 23 representative *Bartonella* strains were selected for further characterization. The cell division protein (*ftsZ*) gene was amplified using the BaftsZF and BaftsZR primers as shown [34]. The primers used for 16S-23S intergenic spacer region (ITS) gene amplification were as shown [35]. The PCR conditions were as described previously. For amplification of the 60 kDa chaperonin protein (*groEL*) gene, hemi-nested primers were designed in this study (as shown in S1 Table) according to the conserved regions of all available *Bartonella* sequences downloaded from the GenBank Database, amplifying approximately 415 bp region. All the PCR products of the expected size were subjected to DNA sequencing and then analyzed by BLASTn algorithm.

All the sequences recovered in this study have been submitted to the GenBank Database and the Accession Numbers have been assigned (Shown in S2 Table).

### Phylogenetic analysis

The *gltA*, *ftsZ*, *groEL*, and ITS gene sequences, as well as those from known *Bartonella* strains deposited in the GenBank database, were manually aligned using ClustalW method within MEGA7.0 and trimmed using SeqMan software (DNASTAR Inc., Madison, USA) to remove poor quality sequences [36]. A phylogeny was constructed based on the nucleotide sequences using the maximum-likelihood (ML) method implemented in PhyML software [37]. The best-fit phylogenetic model was determined by the substitution model test. Bootstrap analysis was carried out with 100 re-samplings. All the trees were mid-point rooted. Accession numbers and rodent hosts of the sequences in this study were shown in the trees.

## Results

### Sample collection

From August to September 2021, a total of 110 rodents were collected in suburban areas of three towns (Hunger, Baiyin Chaoketu, and Naomugeng) of Siziwang Banner, Ulanqab City, Inner Mongolia Autonomous Region. Morphological identification confirmed the existence of four rodent species: 67 *Meriones unguiculatus*, 21 *Spermophilus dauricus*, 21 *Eolagurus luteus*, and 1 *Cricetulus barabensis*.

### Detection of *Bartonella* strains and analysis of *gltA* sequences

Based on DNA sequencing and BLASTn alignment of the *gltA* gene (340 bp), a total of 52 rodent samples tested positive for *Bartonella*: 24 of 67 (35.82%) *M*. *unguiculatus*, 10 of 21 (47.62%) *S*. *dauricus*, and 18 of 21 (85.71%) *E*. *luteus* (Table 1). Of those, 23 representative strains whose *gltA* sequences represent genetically distinct genotypes were selected based on their nucleotide sequences for further investigations.

**Table 1:** Prevalence of *Bartonella* based on PCR targeting the *gltA* gene in rodents collected during August-September 2021 from Siziwang Banner, Inner Mongolia.

Rodent species	Address	Prevalence	Subtotal
*Meriones unguiculatus*	Hunger	34.15% (14/41)	35.82% (24/67)
	Naomugeng	20.00% (2/10)	
	Baiyin Chaoketu	33.33% (8/16)	
*Spermophilus dauricus*	Hunger	41.67% (5/12)	47.62% (10/21)
	Baiyin Chaoketu	55.56% (5/9)	
*Eolagurus luteus*	Baiyin Chaoketu	85.71% (18/21)	85.71% (18/21)
*Cricetulus barabensis*	Baiyin Chaoketu	0.00% (0/1)	0.00% (0/1)
Total		47.27% (52/110)	

**Fig 1:**
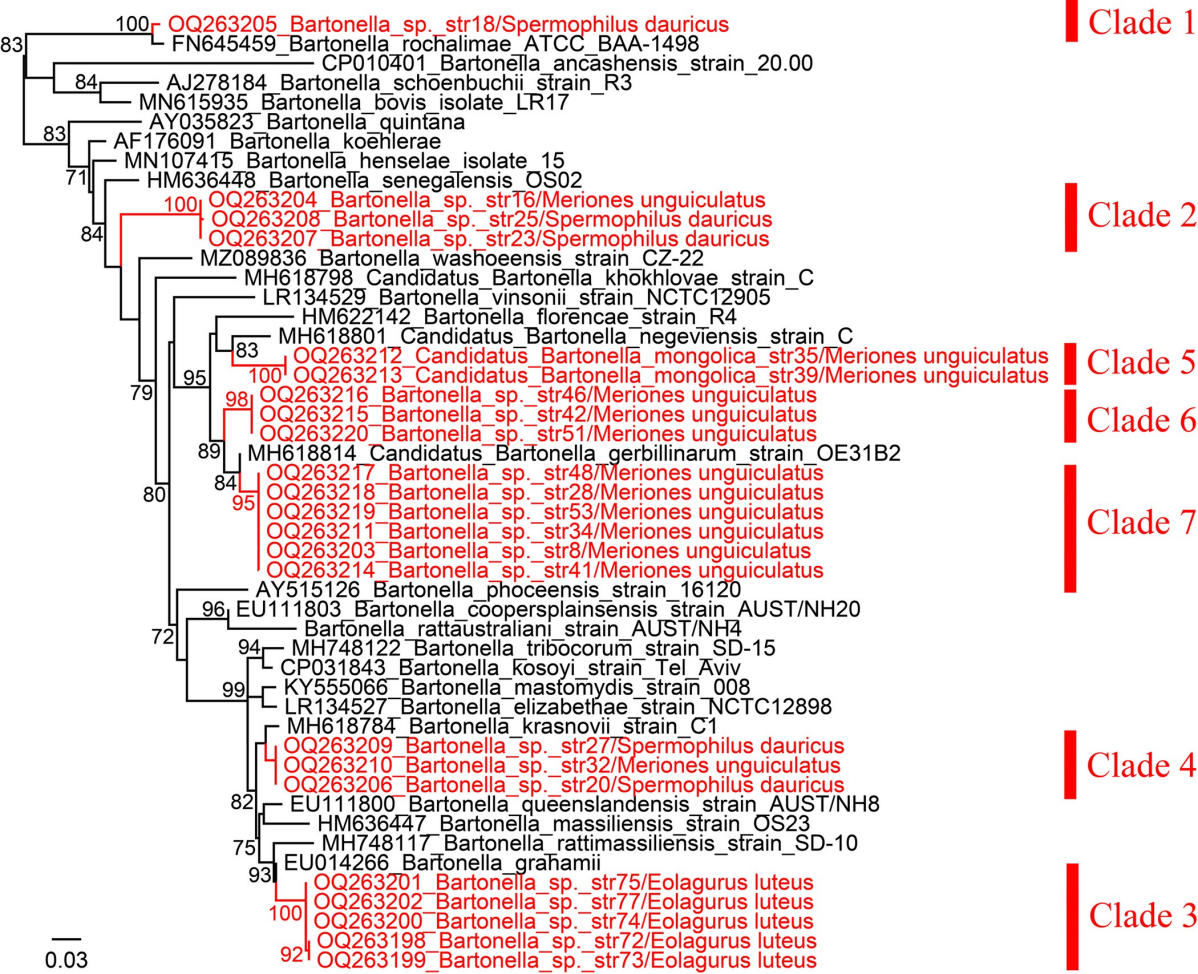
Phylogenetic trees based on the nucleotide sequences of *gltA* gene sequences (340 bp) of *Bartonella* strains using the maximum-likelihood (ML) method. The units of the branch lengths are substitutions per site.

Phylogenetic analysis based on the *gltA* sequences showed that these strains were clearly divided into 7 distinct clades (Fig 1). Genetic analysis indicated clade 1 (strain 18) has the highest similarity to *Bartonella rochalimae*, an emerging zoonotic pathogen, with a nucleotide homology of 98.24%. Clade 2 composed of strains 16, 23, and 25 is mostly related to *Bartonella senegalensis* with identities between 91.18–91.45%. Clade 3 (strains 72, 73, 74, 75, and 77) is only detected in *E*. *luteus* and is closely related to *Bartonella grahamii* (97.06–97.35% nucleotide similarity), a validated human pathogen [11]. For clade 4, all three strains (strains 20, 27, and 32) clustered together with *Bartonella krasnovii*, with an identity of 98.24%. Clades 5, 6, and 7 are all detected in *M*. *unguiculatus*. Clade 5 comprising Strains 35 and 39 is mostly related to *Candidatus* Bartonella negeviensis, with nucleotide similarity as low as 92.94%. Clade 6 (strains 42, 46, and 51) and clade 7 (strains 8, 28, 34, 41, 48, and 53) are both closely related to *Candidatus* Bartonella gerbillinarum, with the highest identities of 96.18% and 97.65%, respectively (Table 2).

**Table 2:** Nucleotide identity of the representative *Bartonella* sequences obtained in this study compared to reference sequences in GenBank.

Clade	Strains	*gltA*	*ftsZ*	ITS	*groEL*
1	18	98.24% *Bartonella rochalimae*	NA	NA	98.55% *artonella washoensis*
2	16, 23, 25	91.18–91.45% *Bartonella senegalensis*	97.20% *Bartonella washoensis*	99.24–99.75% *Bartonella washoensis*	98.55–98.79% *Bartonella washoensis*
3	72, 73, 74, 75, 77	97.06–97.35% *Bartonella grahamii*	96.13% *Bartonella grahamii*	89.79% *Bartonella grahamii*	96.63–97.11% *Bartonella grahamii*
4	20, 27, 32	98.24% *Bartonella krasnovii*	95.74% *Bartonella krasnovii*	90.08–91.94% *Bartonella krasnovii*	95.91–96.15% (strains 20 and 27) *Bartonella grahamii* 95.18% (strain 32) *Bartonella elizabethae*
5	35, 39	92.94% *Candidatus* Bartonella negeviensis	87.09% *Bartonella taylorii*	88.89–89.35% *Candidatus* Bartonella negeviensis	94.39–94.63% *Bartonella vinsonii* subsp. arupensis
6	42, 46, 51	96.18% *Candidatus* Bartonella gerbillinarum	86.71% *Bartonella taylorii*	89.78% *Candidatus* Bartonella gerbillinarum	93.95–94.43% *Bartonella vinsonii*
7	8, 28, 34, 41, 48, 53	97.65% *Candidatus* Bartonella gerbillinarum	87.55–87.56% *Bartonella taylorii*	89.03–89.78% *Candidatus* Bartonella gerbillinarum	93.73–94.94% *Bartonella vinsonii* subsp. arupensis

### Analysis of *ftsZ*, ITS, and *groEL* genes

**Fig 2:**
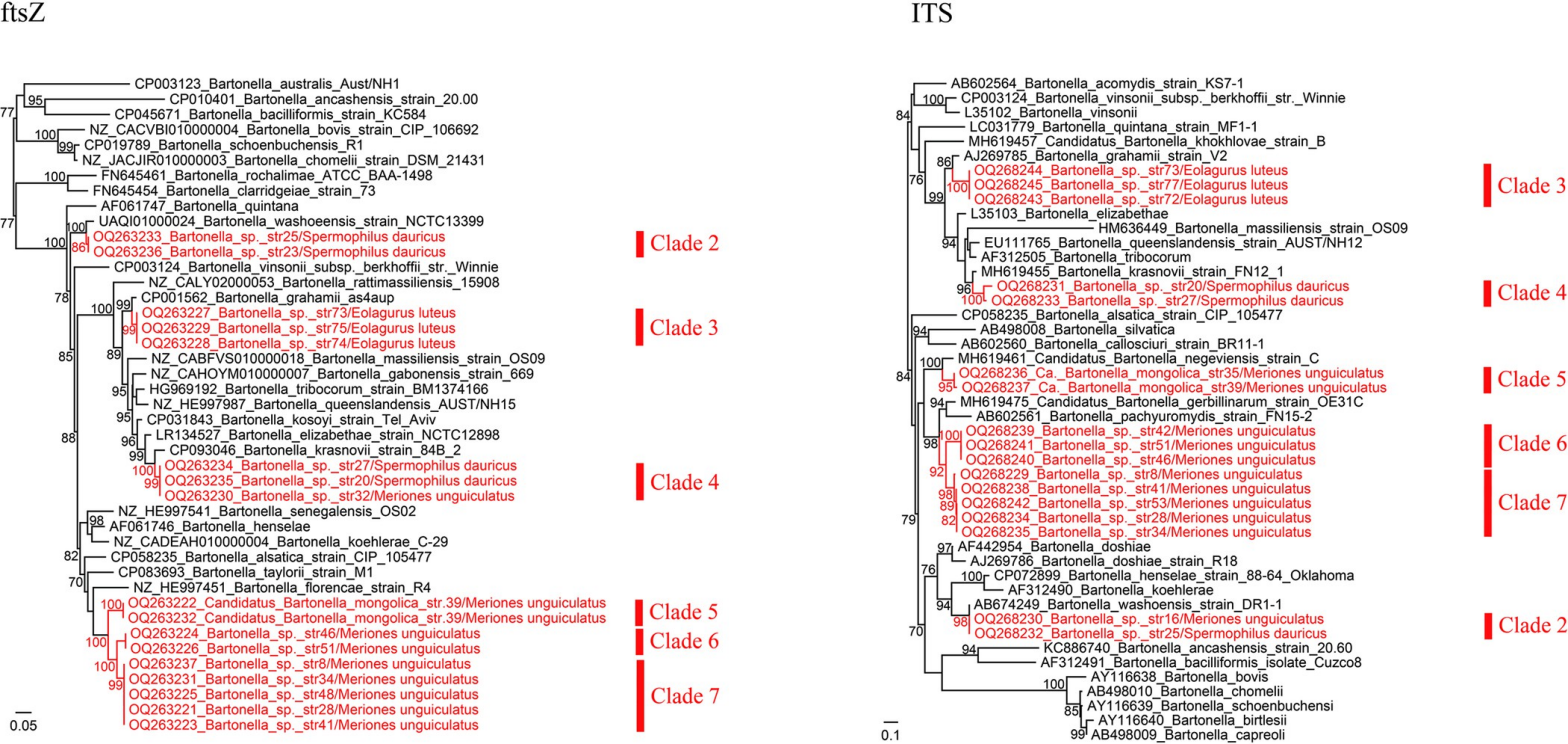
Phylogenetic trees based on the nucleotide sequences of *ftsZ* (818–830 bp) and ITS (394–526 bp) gene sequences of the *Bartonella* strains using the maximum-likelihood (ML) method. The units of the branch lengths are substitutions per site.

For further characterization of the detected *Bartonella* strains, the sequences of *ftsZ* (818–830 bp), ITS (394–526 bp), and *groEL* (415 bp) genes were successfully obtained from representative strains of all 7 clades (Clade 1: 1 strain, Clade 2: 3 strains, Clade 3: 5 strains, Clade 4: 3 strains; Clade 5: 2 strains, Clade 6: 3 strains, Clade 7: 6 strains), except that the *ftsZ* and ITS sequences of Clade 1 (strain 18) are unavailable, probably due to the limited universality of primers. Interestingly, phylogenetic trees based on *ftsZ* and ITS genes are both well consistent with the tree based on the *gltA* gene (Fig 2). Namely, all the strains belonging to certain clades in the *gltA* tree are still clustered together in these phylogenetic trees. To be noticed, clades 5, 6, and 7 are all located in a major clade in phylogenetic trees based on *gltA*, *ftsZ*, and ITS genes.

For the *ftsZ* gene, Clades 2, 3, and 4 showed the highest nucleotide homologies with *B*. *washoensis* (97.20%), *B*. *grahamii* (96.13%), and *B*. *krasnovii* (95.74%), respectively. Clades 5, 6, and 7 are all mostly related to *B*. *taylorii*, with the highest nucleotide similarities of 87.09%, 86.71%, and 87.55–87.56%, respectively (Table 2). As to the ITS gene, clades 2, 3, 4, and 5 share 99.24–99.75% with *Bartonella washoensis*, 89.79% with *B*. *grahamii*, 90.08–91.94% with *B*. *krasnovii*, and 88.89–89.35% to *Candidatus* Bartonella negeviensis, respectively, while Clades 6 and 7 are both mostly related to *Candidatus* Bartonella gerbillinarum (identities 89.78% and 89.03–89.78%, respectively).

**Fig 3:**
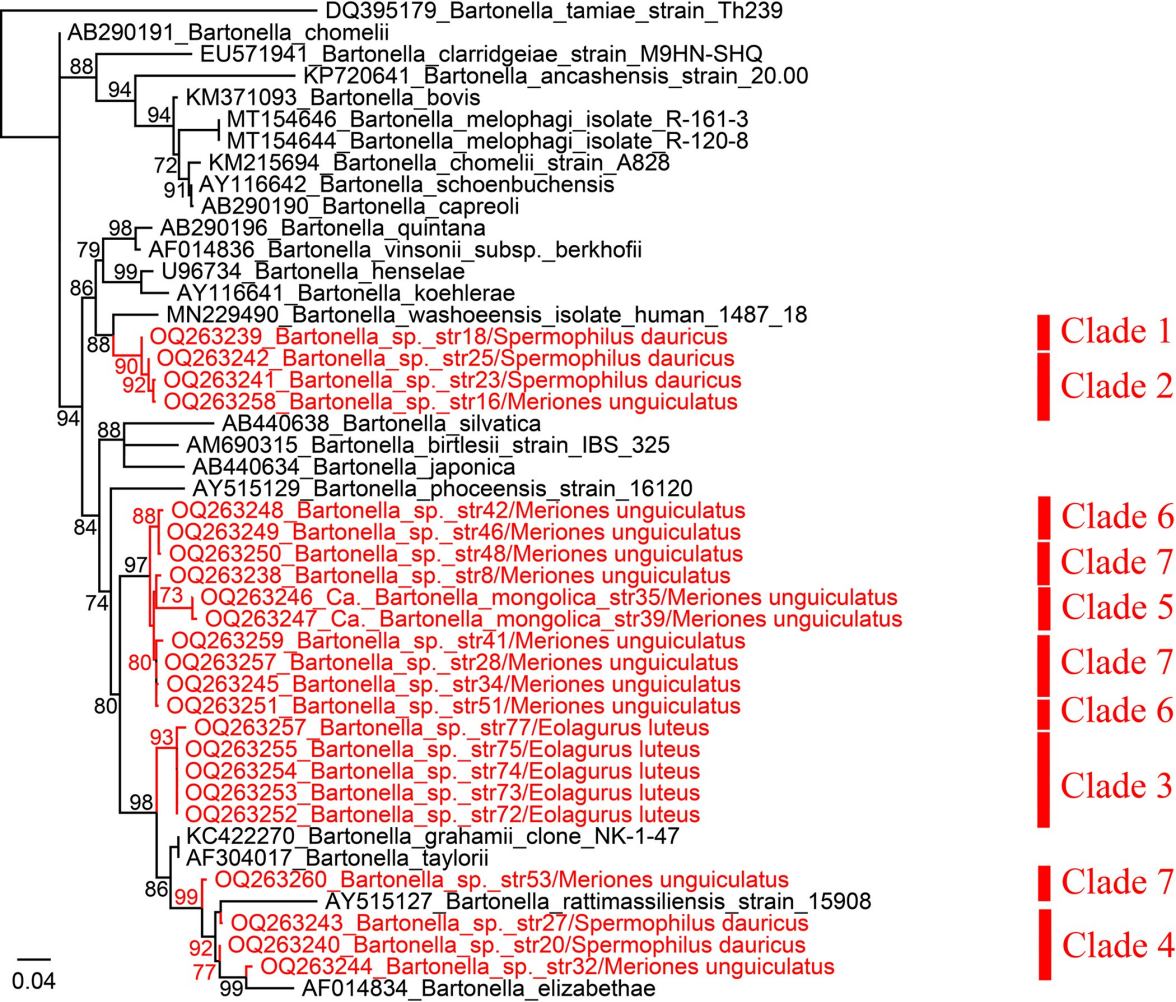
Phylogenetic trees based on the nucleotide sequences of *groEL* gene sequences (415 bp) of *Bartonella* strains using the maximum-likelihood (ML) method. The units of the branch lengths are substitutions per site.

Unexpectedly, the phylogenetic tree based on *groEL* sequences showed that the positions of *Bartonella* strains are obviously inconsistent with *gltA*, *ftsZ*, and ITS trees (Fig 3). Clade 1 (strain 18) and clade 2 (strains 16, 23, and 25) clustered together. Both of them are closely related to *B*. *washoensis* with the identities of 98.55% and 98.55–98.79%. Remarkably, strains from clade 6 (strains 42, 46, and 51) and clade 7 (strains 8, 28, 34, 41, 48, and 53) are dispersed into different lineages. For example, strains 42 and 46 belonging to Clade 6 clustered with strain 48 belonging to clade 7, while strains 28, 34, and 41 (Clade 7) are in the same lineage together with strain 51 belonging to Clade 6. This result may result from several reasons: 1. It is possible that co-infection of different *Bartonella* strains may present in the rodent blood and the *groEL* primers may have been more sensitive to a minor variant present; 2. Lack of some important reference sequences in the GenBank Database and in the phylogenetic trees; 3. Possible recombination or mutations of the *groEL* gene may exist. Clades 3 and 5 are almost unchanged, and they share highest 96.63–97.11% and 94.39–94.63% to *B*. *grahamii* and *Bartonella vinsonii* subsp. arupensis, respectively (Table 2). According to the criteria for *Bartonella* species definition reported in the previous study [32], we suppose that clade 5 undoubtedly represents a novel species. Herein we name it “*Candidatus* Bartonella mongolica” based on the area where it was first detected.

## Discussion

*Bartonella* are considered emerging zoonotic causative agents distributed worldwide. Small mammals such as rodents and bats are the primary hosts of *Bartonella*, and approximately two-thirds of currently reported *Bartonella* species are harbored by rodents. Over the past decades, an increasing number of *Bartonella* species has been reported worldwide. However, despite the species abundance of rodents in China (more than 200 species), the geographical distribution and genetic diversity of rodent-borne *Bartonella* in many areas are still pending.

In this study, a high prevalence of *Bartonella* spp. was observed in rodents from Inner Mongolia, with an overall positive rate of 47.27% (52/110). This rate is similar to those previously reported in rodents from Yunnan Province (43.5%) [38] and Shanxi Province (49.52%) [39]. Three of the four rodent species tested positive for *Bartonella*, with the positive rate varying from 35.82%-85.71%. As is currently known, this may be the first report that *M*. *unguiculatus* and *E*. *luteus* harboring *Bartonella*, which may expand our knowledge on the host range of *Bartonella*. Furthermore, the host specificity of *Bartonella* was also observed in this study. For example, all strains belonging to Clade 3 were detected in *E*. *luteus*, which only account for 19.09% (21/110) of the rodent samples. This is well consistent with previous reports that each *Bartonella* specie only infects one or a few rodent species [40]. Notably, Siziwang Banner is an epidemic area of plague. In this area, *M*. *unguiculatus* and *S*. *dauricus* are the main hosts of *Yersinia pestis*, the etiologic agent of plague [41]. As these rodents live in proximity to humans (mostly herdsmen) in this area, human-rodent interactions are supposed to be frequent. The high infection rate of *Bartonella* in these rodents makes it possible that *Bartonella* spp. may be transmitted to human beings in a similar pathway as plague. Bartonellosis such as cat scratch disease caused by *B*. *henselae* has been widely reported in multiple provinces of China, such as Hebei, Fujian, Anhui, Zhejiang, Guangdong, Hubei, Guizhou, and Beijing [42]. Meanwhile, human cases infected by other *Bartonella* species were also reported including *Bartonella vinsonii* subsp. berkhoffii, *B*. *quintana*, and *B*. *coopersplainsensis* [42–44]. Nonetheless, clinical cases of bartonellosis in Inner Mongolia have never been reported, probably due to minor symptoms (fever, malaise, etc.) and overlap with other infectious diseases. It is plausible to suppose that the risk of human infection by *Bartonella* is largely underestimated or ignored.

In this study, remarkable genetic diversity of *Bartonella* was observed, with seven distinct phylogenetic clades identified. Of those, the *gltA*, *ftsZ*, ITS, and *groEL* genes of Clade 5 showed the highest nucleotide identities of 92.94%, 87.09%, 88.89–89.35%, and 94.39–94.64% to reported species. According to the criteria for the definition of *Bartonella* species [31], these remarkable genetic divergences suggest that they represent a novel species. Herein we name it “*Candidatus* Bartonella mongolica”. Genetic and phylogenetic analysis indicated that all the key genes of clade 3 strains are closely related to *B*. *grahamii*. Namely, this clade should represent a variant of *B*. *grahamii*. As a validated human pathogen, *B*. *grahamii* has been reported to cause cat scratch disease, with the symptoms of neuroretinitis and enlarged lymph nodes [11]. The high positive rate of *B*. *grahamii* in *E*. *luteus* and its potential human pathogenicity may suggest the risk of human infection.

### Ethics approval and consent to participate

This study protocol was approved by the Ethics Committee of the National Institute for Communicable Disease Control and Prevention, the Chinese CDC (Approval No. 2021–011), and the General Center for Disease Control and Prevention of the Inner Mongolia Autonomous Region. All the rodents were treated in a humane manner in accordance with the “Rules for Implementation of Laboratory Animal Medicine” from the National Health Commission, China.

### Availability of data and materials

All sequence files are available from the GenBank database (Accession Numbers are shown in S2 Table).

## Supporting information

S1 Table The primers used for amplification of the *groEL* gene from *Bartonella* strains by hemi-nested PCR. (DOCX)

S2 Table Genbank numbers of the *gltA*, *ftsZ*, *groEL*, and ITS sequences of *Bartonella* strains in this study. (DOCX)
