# Supplementary material for: The diverse genetic genotypes of Bartonella species circulating in rodents from Inner Mongolia, Northern China
Source: PLoS Negl Trop Dis. 2023 Jun 29;17(6):e0011462. doi: 10.1371/journal.pntd.0011462 (PMC10337887; doi:10.1371/journal.pntd.0011462)
Supplement: S1 Table — (DOCX) [file pntd.0011462.s001.docx]

Table S1. The primers used for amplification of the *groEL* gene from *Bartonella* strains by hemi-nested PCR.

| Primer | Cycle | Sequence | Expected length |
| --- | --- | --- | --- |
| BartEL-ex5 | 1 | 5-TTRGAAGTYGTGGAAGGWATG-3 | 450 bp |
| BartEL-in5 | 2 | 5-CCTTAYTTYGTCACMAATGCT-3 |  |
| BartEL-3 | 1, 2 | 5-RTCATARTCAGAAGTTGTTTCTTC-3 |  |
