# Supplementary material for: The diverse genetic genotypes of Bartonella species circulating in rodents from Inner Mongolia, Northern China
Source: PLoS Negl Trop Dis. 2023 Jun 29;17(6):e0011462. doi: 10.1371/journal.pntd.0011462 (PMC10337887; doi:10.1371/journal.pntd.0011462)
Supplement: S2 Table — (DOCX) [file pntd.0011462.s002.docx]

Table S2. Genbank numbers of the *gltA*, *ftsZ*, *groEL*, and ITS sequences of *Bartonella* strains in this study.

| No. | **Gene** | **Genbank Number** | **Bacterial strain** |
| --- | --- | --- | --- |
| 1 | *gltA* | OQ263198 | *Bartonella* sp. str.72 |
| 2 | *gltA* | OQ263199 | *Bartonella* sp. str.73 |
| 3 | *gltA* | OQ263200 | *Bartonella* sp. str.74 |
| 4 | *gltA* | OQ263201 | *Bartonella* sp. str.75 |
| 5 | *gltA* | OQ263202 | *Bartonella* sp. str.77 |
| 6 | *gltA* | OQ263203 | *Bartonella* sp. str.8 |
| 7 | *gltA* | OQ263204 | *Bartonella* sp. str.16 |
| 8 | *gltA* | OQ263205 | *Bartonella* sp. str.18 |
| 9 | *gltA* | OQ263206 | *Bartonella* sp. str.20 |
| 10 | *gltA* | OQ263207 | *Bartonella* sp. str.23 |
| 11 | *gltA* | OQ263208 | *Bartonella* sp. str.25 |
| 12 | *gltA* | OQ263209 | *Bartonella* sp. str.27 |
| 13 | *gltA* | OQ263210 | *Bartonella* sp. str.32 |
| 14 | *gltA* | OQ263211 | *Bartonella* sp. str.34 |
| 15 | *gltA* | OQ263212 | *Candidatus* Bartonella mongolica str.35 |
| 16 | *gltA* | OQ263213 | *Candidatus* Bartonella mongolica str.39 |
| 17 | *gltA* | OQ263214 | *Bartonella* sp. str.41 |
| 18 | *gltA* | OQ263215 | *Bartonella* sp. str.42 |
| 19 | *gltA* | OQ263216 | *Bartonella* sp. str.46 |
| 20 | *gltA* | OQ263217 | *Bartonella* sp. str.48 |
| 21 | *gltA* | OQ263218 | *Bartonella* sp. str.28 |
| 22 | *gltA* | OQ263219 | *Bartonella* sp. str.53 |
| 23 | *gltA* | OQ263220 | *Bartonella* sp. str.51 |
| 24 | *ftsZ* | OQ263221 | *Bartonella* sp. str.28 |
| 25 | *ftsZ* | OQ263222 | *Candidatus* Bartonella mongolica str.39 |
| 26 | *ftsZ* | OQ263223 | *Bartonella* sp. str.41 |
| 27 | *ftsZ* | OQ263224 | *Bartonella* sp. str.46 |
| 28 | *ftsZ* | OQ263225 | *Bartonella* sp. str.48 |
| 29 | *ftsZ* | OQ263226 | *Bartonella* sp. str.51 |
| 30 | *ftsZ* | OQ263227 | *Bartonella* sp. str.73 |
| 31 | *ftsZ* | OQ263228 | *Bartonella* sp. str.74 |
| 32 | *ftsZ* | OQ263229 | *Bartonella* sp. str.75 |
| 33 | *ftsZ* | OQ263230 | *Bartonella* sp. str.32 |
| 34 | *ftsZ* | OQ263231 | *Bartonella* sp. str.34 |
| 35 | *ftsZ* | OQ263232 | *Candidatus* Bartonella mongolica str.35 |
| 36 | *ftsZ* | OQ263233 | *Bartonella* sp. str.25 |
| 37 | *ftsZ* | OQ263234 | *Bartonella* sp. str.27 |
| 38 | *ftsZ* | OQ263235 | *Bartonella* sp. str.20 |
| 39 | *ftsZ* | OQ263236 | *Bartonella* sp. str.23 |
| 40 | *ftsZ* | OQ263237 | *Bartonella* sp. str.8 |
| 41 | *groEL* | OQ263238 | *Bartonella* sp. str.8 |
| 42 | *groEL* | OQ263239 | *Bartonella* sp. str.18 |
| 43 | *groEL* | OQ263240 | *Bartonella* sp. str.20 |
| 44 | *groEL* | OQ263241 | *Bartonella* sp. str.23 |
| 45 | *groEL* | OQ263242 | *Bartonella* sp. str.25 |
| 46 | *groEL* | OQ263243 | *Bartonella* sp. str.27 |
| 47 | *groEL* | OQ263244 | *Bartonella* sp. str.32 |
| 48 | *groEL* | OQ263245 | *Bartonella* sp. str.34 |
| 49 | *groEL* | OQ263246 | *Candidatus* Bartonella mongolica str.35 |
| 50 | *groEL* | OQ263247 | *Candidatus* Bartonella mongolica str.39 |
| 51 | *groEL* | OQ263248 | *Bartonella* sp. str.42 |
| 52 | *groEL* | OQ263249 | *Bartonella* sp. str.46 |
| 53 | *groEL* | OQ263250 | *Bartonella* sp. str.48 |
| 54 | *groEL* | OQ263251 | *Bartonella* sp. str.51 |
| 55 | *groEL* | OQ263252 | *Bartonella* sp. str.72 |
| 56 | *groEL* | OQ263253 | *Bartonella* sp. str.73 |
| 57 | *groEL* | OQ263254 | *Bartonella* sp. str.74 |
| 58 | *groEL* | OQ263255 | *Bartonella* sp. str.75 |
| 59 | *groEL* | OQ263256 | *Bartonella* sp. str.77 |
| 60 | *groEL* | OQ263257 | *Bartonella* sp. str.28 |
| 61 | *groEL* | OQ263258 | *Bartonella* sp. str.16 |
| 62 | *groEL* | OQ263259 | *Bartonella* sp. str.41 |
| 63 | *groEL* | OQ263260 | *Bartonella* sp. str.53 |
| 64 | ITS | OQ268229 | *Bartonella* sp. str.8 |
| 65 | ITS | OQ268230 | *Bartonella* sp. str.16 |
| 66 | ITS | OQ268231 | *Bartonella* sp. str.20 |
| 67 | ITS | OQ268232 | *Bartonella* sp. str.25 |
| 68 | ITS | OQ268233 | *Bartonella* sp. str.27 |
| 69 | ITS | OQ268234 | *Bartonella* sp. str.28 |
| 70 | ITS | OQ268235 | *Bartonella* sp. str.34 |
| 71 | ITS | OQ268236 | *Candidatus* Bartonella mongolica str.35 |
| 72 | ITS | OQ268237 | *Candidatus* Bartonella mongolica str.39 |
| 73 | ITS | OQ268238 | *Bartonella* sp. str.41 |
| 74 | ITS | OQ268239 | *Bartonella* sp. str.42 |
| 75 | ITS | OQ268240 | *Bartonella* sp. str.46 |
| 76 | ITS | OQ268241 | *Bartonella* sp. str.51 |
| 77 | ITS | OQ268242 | *Bartonella* sp. str.53 |
| 78 | ITS | OQ268243 | *Bartonella* sp. str.72 |
| 79 | ITS | OQ268244 | *Bartonella* sp. str.73 |
| 80 | ITS | OQ268245 | *Bartonella* sp. str.77 |
